# Gender differences and factors affecting opinions on condom use and sexual experiences among adolescents in a high teenage pregnancy setting in the Volta Region, Ghana

**DOI:** 10.1186/s12978-025-01990-7

**Published:** 2025-05-31

**Authors:** Desmond Klu, Percival Delali Agordoh, Charles Azagba, Evelyn Acquah, Phidelia Doegah, Alfred Kwesi Manyeh, Evelyn Korkor Ansah, Margaret Gyapong

**Affiliations:** 1 Institute of Health Research, University of Health and Allied Sciences, Ho, Volta Region Ghana; 2 School of Allied Health Sciences, University of Health and Allied Sciences, Ho, Volta Region Ghana; 3 Hohoe Municipal Health Directorate, Ghana Health Service, Hohoe, Volta Region Ghana

**Keywords:** Gender, Adolescents, Condom, Sexual, Health, Ghana

## Abstract

**Background:**

Gender is crucial in understanding the sexual behaviour of adolescents regarding their condom use and sexual experiences. However, not much is known about the role gender plays in adolescents’ opinions on condom use and their sexual experiences and other factors that influence these opinions in high-adolescent pregnancy settings in rural Ghana. This study sought to examine the gendered dynamics and factors that shape the opinions of adolescent girls and boys on condom use and how that affects their sexual experiences in the high teenage pregnancy setting of Adaklu District, in the Volta region of Ghana.

**Methods:**

Data for this study were extracted from a larger primary baseline cross-sectional study among 188 adolescents (109 girls and 79 boys) aged 10–19 years in 30 communities. Data from a semi-structured questionnaire were analyzed using a bivariate analysis and binary logistic regression.

**Results:**

The results indicate adolescent girls were less likely (aOR = 0.16, CI 0.06–0.43) to agree to the opinion that condoms are an effective way of preventing HIV compared to boys. Adolescent girls are less likely to agree to the opinion that they have been pressured into sex (aOR = 0.15; CI 0.06–0.39), but have higher odds (aOR = 2.58; CI 1.33–1.64) to agree that purchasing condoms is embarrassing for them compared to males. Regarding age, adolescents 10–14 years are less likely (aOR = 0.43; CI 0.19–0.99) to agree that condoms are an effective in preventing HIV compared to those aged 15–19 years. Adolescents aged 10–14 years had higher odds (aOR = 2.91; CI 1.28–6.63) to agree that purchasing condoms is embarrassing for them to compared to the 15–19 year olds. Employment status of adolescents significantly influences their opinion on condom use, where adolescents who are currently employed are more likely to agree to the opinion that it would be embarrassing for them to go and purchase condoms compared to the unemployed ones.

**Conclusions:**

Gender, age and employment status of adolescents are critical to their sexual and reproductive health and wellbeing, as the study findings show distinct sexual experiences of adolescent boys and girls. These important factors should be considered when formulating sexual and reproductive health policies and programs for adolescents to meet their needs.

**Supplementary Information:**

The online version contains supplementary material available at 10.1186/s12978-025-01990-7.

## Plain language summary

Sexual and reproductive health needs and experiences of adolescent boys and girls are different, and these differences influence their ideas and opinions on issues such as condom use and male–female sexual activities. Social constructs associated with the biological identity of adolescents coupled with other individual socio-cultural and economic factors plays a critical role in shaping the opinion of adolescents in settings with high prevalence of teenage pregnancy. However, the role of gender and other factors in influencing adolescents' perceptions of condom use and other sexual experiences has not been adequately explored. This paper, therefore, examines the role of gender and other socio-economic factors that influence the opinions of adolescents on condom use and their heterosexual experiences in a high teenage pregnancy setting in the Volta region of Ghana. The results show that the gender identity of adolescents influences their views on condom use and other sexual experiences. Adolescent girls are more likely to believe that condoms are not effective in the prevention of HIV and are also more likely to consider it embarrassing to go and purchase condoms, when compared to boys. Further, adolescents who are below the age of 15 are more likely to believe that condoms are not effective in preventing HIV and also to consider it embarrassing to purchase condoms compared to those 15 years and above. This highlights the importance of gender stereotyping in shaping opinions of adolescents on condom use and sexual experiences. It also points to the importance of intersectional analysis to understand adolescent behaviours, which can then shape the design of programs and policies to support healthy sexual and reproductive choices for adolescent girls and boys. These perceptions place adolescent girls in a compromising position as far as their sexual and reproductive health is concerned and they are often vulnerable to risky sexual behaviours.

**Supplementary Information:**

The online version contains supplementary material available at 10.1186/s12978-025-01990-7.

## Résumé

**Contexte:**

Le genre est crucial pour comprendre le comportement sexuel des adolescent(e)s en ce qui concerne l’utilisation des préservatifs et les expériences sexuelles. Cependant, on ne sait pas grand-chose sur le rôle que joue le genre dans les opinions des adolescent(e)s sur l’utilisation du préservatif et leurs expériences sexuelles et sur d’autres facteurs qui influencent ces opinions dans un contexte à forte prévalence de grossesses chez les adolescentes dans les zones rurales du Ghana. Cette étude visait à examiner les dynamiques de genre et les facteurs qui façonnent les opinions des adolescentes et des adolescents sur l’utilisation du préservatif et la façon dont cela affecte leurs expériences sexuelles dans un contexte à forte prévalence de grossesses chez les adolescentes du district Adaklu, dans la région de la Volta au Ghana.

**Méthodes:**

Les données de cette étude ont été extraites d’une étude transversale de base préliminaire plus large menée auprès de 188 adolescent(e)s (109 filles et 79 garçons) âgé(e)s de 10 à 19 ans dans 30 communautés. Les données d’un questionnaire semi-structuré ont été analysées à l’aide d’une analyse bivariée et d’une régression logistique binaire.

**Résultats:**

Les résultats indiquent que les adolescentes étaient moins susceptibles (aOR (rapport de cotes corrigé) = 0,16, IC (intervalle de confiance = 0,06–0,43) d’être d’accord avec l’opinion selon laquelle les préservatifs sont un moyen efficace de prévention du VIH par rapport aux garçons. Les adolescentes sont moins susceptibles d’être d’accord avec l’opinion selon laquelle elles ont subi des pressions pour avoir des rapports sexuels (aOR = 0,15 ; IC = 0,06–0,39), mais elles sont plus susceptibles (aOR = 2,58 ; IC = 1,33–1,64) de convenir que l’achat de préservatifs est embarrassant pour elles par rapport aux hommes. En ce qui concerne l’âge, les adolescent(e)s de 10 à 14 ans sont moins susceptibles (aOR = 0,43 ; IC = 0,19–0,99) de convenir que les préservatifs sont un moyen efficace de prévention du VIH par rapport à ceux et celles âgé(e)s de 15 à 19 ans. Les adolescent(e)s âgé(e)s de 10 à 14 ans étaient plus susceptibles (aOR = 2,91 ; IC = 1,28–6,63) de convenir que l’achat de préservatifs est embarrassant pour eux(elles) par rapport aux 15–19 ans. La situation d’emploi des adolescents influence considérablement leur opinion sur l’utilisation du préservatif, où les adolescents qui ont actuellement un emploi sont plus susceptibles d’être d’accord avec l’opinion selon laquelle il serait embarrassant pour eux d’aller acheter des préservatifs par rapport à ceux qui sont au chômage.

**Conclusion:**

Le sexe, l’âge et la situation d’emploi des adolescents sont essentiels à leur santé sexuelle et reproductive ainsi qu’à leur bien-être, car les résultats de l’étude montrent des expériences sexuelles distinctes chez les garçons et les filles. Ces facteurs importants doivent être pris en compte lors de la formulation de politiques et de programmes de santé sexuelle et reproductive pour les adolescent(e)s afin de répondre à leurs besoins.

## Background

Teenage pregnancy is an important public health problem throughout the world, with long-term consequences for the health and well-being of mothers and children [1–4]. Between 2016 and 2020, Ghana recorded 542,131 pregnancies amongst adolescent girls aged 15–19 years and 13,444 pregnancies amongst young teenagers aged 10–14 years according to the Ghana Health Service District Health Information Management Health System (DHIMS). Further, there has been an increase in teenage pregnancy prevalence between 2014 and 2022 from 14 to 15%. Studies especially in sub-Saharan Africa (SSA), have revealed some socio-cultural norms and practices that influence and compel adolescent girls either to engage in risky sexual behaviours which lead to pregnancy or that forced into marrying early [5–7]. For example, a study by Dubik and colleagues [5], found that, being from polygamous families, parents’ desire for grandchildren, multiple sexual partners, poverty, peer pressure, social prestige associated with high fertility and marriage were socio-cultural factors affecting teenage pregnancy and child marriage in the Mamprusi municipality in Ghana. One potential solution to reducing the rates of unwanted pregnancy and sexually transmitted infections (STDs) among teenagers aged 10 to 19 years is the promotion of safe sex practices, such as condom use [8–11]. However, studies show that the use of condoms varies between males and females; with the former using condoms more frequently than the latter [12–15]. This may reflect differences in the levels of control males and females exert over sexual experiences and differences in risk and vulnerability perceptions [16, 17].

Research has shown that gender differences in condom use and opinions among adolescents are influenced by a range of individual, social, and cultural factors [18–22]. For instance, a study by Shearer and colleagues [21] found that young men may be more likely to engage in risky sexual behaviors, such as unprotected sex, due to societal expectations of masculinity and sexual prowess. Further, a study by Olaniran and colleagues [22] found that gender played a significant role in perceptions and attitudes towards condom use, with females often at a disadvantage in negotiating safe sex practices as a result of socio-economic constraints and gender discrimination. Young women may face social pressure to prioritize the desires of their male partners over their own needs and preferences, which could lead to a reluctance to use condoms or negotiate condom use. Other studies revealed that males tend to have more positive attitudes toward condom use compared to females [19, 20]. Gender norms place greater responsibility for contraception on females, leading to perceptions that condom use is primarily a female responsibility [23–25].

Thus, gender is crucial in understanding the sexual behaviours of adolescents regarding their condom use and sexual experiences [12, 26, 27]. It plays a critical role in the sexual health needs and experiences of adolescents due to their biological differences and socially expected roles. There are also gender differences in the rates of adolescent risky sexual behaviours and the receipt of preventive care. Studies revealed that adolescent males are more likely to engage in risky sexual behaviours and have lower rates of sexual preventive care than females [28–31]. Irrespective of the underlying opinions, these gender differences in attitudes towards condom use and sexual experiences can have significant implications for the rates of adolescent pregnancy and sexually transmitted infections. Gender differences in opinions on condom use and sexual experiences-influenced by gender relations and gender norms may influence the effectiveness of condom use. Studies have also explored factors influencing adolescents’ opinion on condom use and sexual experiences [22, 24, 32]. They found adequate sex education, unplanned sexual intercourse, past condom use behaviour, fears of teenage pregnancy and sexually transmitted diseases (STDs) as factors influencing adolescents' opinions (both in favour and in opposition) on the use of condoms and sexual activities. Meanwhile, for adolescents’ sexual reproductive health policies and public health interventions to be effective, the unique needs, perspectives and lived experiences of both female and male adolescents must be considered to address the barriers that prevent them from accessing and using condoms effectively. An implementation gap exists in Ghana’s Adolescent Health Service Policy and strategy (2016–2020) concerning age and gender-specific needs and interventions for adolescents to enable them make informed SRH decision.

A number of studies have examined sexual and reproductive issues affecting adolescents residing in high teenage pregnancies setting in rural Ghana and Volta region [33–36]. These studies specifically examine factors influencing poor communication of sexual issues between parents and adolescents [33], adolescent perception of their sexual and reproductive health rights and access to SRH information and services [34], SRH knowledge of adolescents [35] and association between social capital and utilisation of SRH service [36].

However, not much is known about the role gender plays in adolescents’ opinions on condom use and their sexual experiences and other factors that influence these opinions in high-adolescent pregnancy settings in rural Ghana such as the Adaklu district. In this district, located in the Volta region of Ghana, the teenage pregnancy rate was measured as high as 23.3% in 2015 according to data from the District Health Information Management System.

The objective of the study was to explore gender dynamics and socio-cultural factors that influence the opinions on condom use and sexual experiences of adolescents in a high-adolescent pregnancy setting of Adaklu district in the Volta Region of Ghana.

## Methods

### Study design

Data for this study were extracted from a larger phase one pre-intervention and exploratory set of activities, involving a primary baseline mixed methods cross-sectional study among adolescents (10–19 years), their parents/caregivers, community leaders, and community health providers. The larger study, which adopted an implementation research approach, collected data on sexual and reproductive health issues such as knowledge and attitudes toward contraceptive use, teenage pregnancy, HIV/AIDS and other sexually transmitted diseases (STDs), sexuality, gender and social norms in order to obtain a better understanding of the reasons for the high teenage pregnancy rates and why communities within the district mistrust the implementation of adolescent health clubs to reduce teenage pregnancy. However, the data extracted for this study from the larger data was limited to an aspect of the quantitative section on socio-demographic characteristics of adolescents, gender characteristics and statements measuring their opinion on condom use and other heterosexual experiences.

### Study area and period

This study was conducted in Adaklu district, Volta Region of Ghana, in November 2019 and the district consists of 91 communities. Based on the 2021 Population and Housing census, the district has an estimated population of 38,649, made up of 18,963 males and 19,686 females, representing 49.1% and 50.9%, respectively. The estimated adolescent population (10–19 year olds) in the district is 9887. The age-dependency ratio in the district is 72.1, the total fertility rate of the district is 2.4, and the crude birth rate is 18.3. The district has an estimated 37,904 household population with an average household size of 3.7 persons per house, higher than the regional average of 3.3 [37]. The study area is strictly a patriarchal society where both men and women are socially expected to perform some roles and responsibilities and adhere to behaviours influenced by customary and traditional norms and values [38].

### Study population

The study respondents were adolescent boys and girls aged 10–19 years from 30 selected communities in the district who lived in the area for a minimum of six months.

### Sampling technique and sample size

A total of 30 communities (clusters) with a minimum population of 500 residents were randomly selected in the district out of the 91 communities in the district which represent one-quarter of the communities in the study area. The modified Expanded Program of Immunisation (EPI) cluster sampling technique was used to select seven households in each community and within these households’ questionnaires were administered to adolescents (10–19 years). A conscious effort was made to include an equal number of male and female adolescents who were members or non-members of adolescent health clubs. The process for selecting adolescents involved several steps. First the centre of the selected community was located and a bottle was spun to determine the direction for selection. Starting from the fifth house in the indicated direction, field officers identified households with eligible adolescents. If multiple eligible adolescents were present, a random selection was made through a ballot. If no eligible adolescents were found, field officers moved to every fifth house until all were exhausted then repeated the process in the opposite direction, and then in the remaining two directions. Questionnaires were administered to adolescents aged 10–19 years in these households. Efforts were made to ensure an equal representation of male and female respondents. If a community only had female adolescents, field officers were instructed to select only male adolescents from the next community to balance the gender representation. This strategy ensured a fair and balanced assessment of the study’s subject matter. The details of this selection process have already been published [34].

The sample size for the larger study was calculated using the single population proportion formulation under the following axioms: 17.6% proportion; confidence level was taken to be 95% with α = 0.05 value, 3% margin of error and a design effect of 2 and 5% were added for the expected non-response rate, constituting a final sample size of 221 adolescents. Based on the outcome variables, the total sample size of adolescents used was 188 adolescents, comprising 109 females and 79 males. Frequencies, percentages and chi-square tests were used to achieve the study objective.

### Data collection tools and procedures

Baseline data were collected over a month from October to November 2019. A structured questionnaire was developed and administered to the respondents. The development of the questionnaire was guided by previous adolescent sexual and reproductive health studies in West and Central Africa [6, 38–40].

The main sections included in the questionnaire were sociodemographic characteristics, sexual and reproductive health knowledge and services, sexuality, condom use and contraception, risky sexual behavior, sexual harassment, and coercion (See Additional file 1). Data collection was conducted for 30 days by seven trained research assistants. These research assistants were between the ages 20–25 years, comprising four males and three females. The research assistants were recruited based on their skills and proficiency in English and Ewe (a widely spoken language in the communities). The questionnaire was pre-tested, and all the necessary corrections made.

### Measurements

#### Dependent variables

The dependent variables in this study were adolescents’ opinions on condom use and their heterosexual experiences. The following statements were used to measure their opinion on condom use:

i. *Condoms are an effective method for preventing pregnancy*
ii. *A condom can be used more than once*
iii. *A girl can suggest to her boyfriend that he uses a condom*
iv. *A boy can suggest to his girlfriend that he uses a condom*
v. *Condoms are an effective way of protecting against HIV/AIDS*
vi. *It would be too embarrassing for someone like me to buy or obtain condoms*
vii. *If a girl suggested using condoms to her partner, it would mean that she didn’t trust him*
viii. *Condoms reduce sexual pleasure*
ix. *Condoms can slip off the man and disappear inside the woman’s body*
x. *If unmarried couples want to have sexual intercourse before marriage, they should use condoms*

All the ten statements used in measuring opinion on condom use among adolescent males and females had three responses: agree, disagree and ‘don’t know’. In the context of this study, heterosexual experiences refer to personal sexual experiences and experiments adolescent girls and boys have ever engaged in with the opposite sex. The following statements were employed to measure adolescents’ heterosexual experiences:

i. *Physical contact-Kissing and hugging*
ii. *Transactional sex-pay money for sex*
iii. *Transactional sex-receive money or gifts for sex*
iv. *Adolescent pressured to have sex*
v. *Sexually Coerced*
vi. *Sexually harassed*
vii. *Ever use all forms of contraceptives*

All six statements had a “yes” or “no” response to each statement.

#### Independent variables

The main independent variable was the gender of adolescents, which was measured as ‘boy’ or ‘girl’. In the scope of this study, gender is considered as male sex (boy) and female sex (girl) with reference to their heterosexual roles, experiences and perspectives on condom use as a means to prevent pregnancy and STIs including HIV/AIDS.

Other predictor variables considered were the demographic and socio-cultural background of adolescent boys and girls such as age (10–14 and 15–19), religion (Christian and Atheist), educational level attainment (none, primary and secondary), current schooling status (attending and not attending) and living arrangement (living with father only, living with mother only, living with both parents). Employment status (employed and unemployed, adolescent club membership (member and non-member), attendance of social events (yes and no) and discussion of sexual matters with father and mother (yes and no) were other predictor variables considered in this study.

### Ethical approval

This study received ethical approval from the Research Ethics Committee (REC) of the University of Health and Allied Sciences (UHAS), with the reference number *‘UHAS-REC A.8 *[3]* 18–19’.* All adult respondents who took part in the study signed a written informed consent form, indicating their willingness and agreement to participate in this research. All methods used were carried out in accordance with relevant guidelines and procedures. For respondents less than 18 years of age (minors), written consent was obtained from their respective parents or caregivers and assent from the minors before they were interviewed. All respondents were informed of their right to withdraw from the study at any time. The study also gave each respondent a unique identifier, which ensured the privacy and confidentiality of their responses.

### Data processing and analysis

The data collected was systematically coded and entered into REDCap, ensuring quality through legal values, range checks and validation rules. After cleaning for internal consistency, the data was exported to SPSS Version 25 for analysis. Frequency distributions and inferential statistics were calculated using design-based analysis with percentages and cross-tabulations. Bivariate analysis assessed the association between the outcome and the main predictor variable, determining statistical significance. Three binary logistic regression models were developed: the first regressed gender and other predictors on adolescents’ views on condom use for HIV prevention; the second examined the relationship between gender, other predictors and the opinion that purchasing condoms is embarrassing; the third analysed the relationship between gender, other factors and sexual experiences among adolescents. Similar analytical technique with details was used by Klu et al., [34].

## Results

### Description of background characteristics

The results from Table 1 showed that a higher proportion of adolescent boys (67.1%) were within the age bracket of 15–19 years relative to adolescent girls (60.1%). On the other hand, among 10 to 14-year-olds, adolescent girls constitute the highest proportion (39.9%). An almost equal proportion of adolescent girls (98.9%) and boys (97.5%) were Christians. More adolescent boys (50.6%) had attained secondary education compared with adolescent girls (44.7%); a higher proportion (16.0%) of adolescent girls had no formal education relative to 11.4% of adolescent boys; and more adolescent girls (10.1%) were currently not in school compared to adolescent boys (6.3%). A higher percentage (44.3%) of adolescent boys lived with both parents, 42.0% of adolescent girls lived with only their mothers.

**Table 1:** Background characteristics of adolescent males and females. Source: IDRC Adolescent Health Intervention Project, 2019

Characteristics	Adolescent Boys (N = 79)	Adolescent Girls (N = 109)
	n (%)	n (%)
Age		
10–14	26 (32.9)	75 (39.9)
15–19	53 (67.1)	113 (60.1)
Religion		
Christian	77 (97.5)	186 (98.9)
Atheist	2 (2.5)	2 (1.1)
Educational Level		
None	9 (11.4)	30 (16.0)
Primary	30 (38.0)	74 (39.4)
Secondary	40 (50.6)	84 (44.7)
Currently schooling		
Yes	74 (93.7)	169 (89.9)
No	5 (6.3)	19 (10.1)
Living Arrangement		
Living with only father	16 (20.3)	37 (19.7)
Living with only mother	28 (35.4)	79 (42.0)
Living with both parent	35 (44.3)	72 (38.3)
Adolescent Club Member		
No	34 (54.8)	28 (45.2)
Yes	45 (35.7)	81 (64.3)
Social Event Attendance		
No	26 (31.3)	57 (68.7)
Yes	53 (50.5)	52 (49.5)
Employment Status		
Not employed	68 (39.3)	105 (60.7)
Employed	11 (73.3)	4 (26.7)
Discussed sexual issues with Father		
No	71 (44.1)	90 (55.9)
Yes	8 (29.6)	19 (70.4)
Discussed sexual issues with Mother		
No	62 (52.1)	57 (47.9)
Yes	17 (24.6)	52 (75.4)

### Gendered differences in opinion on condom use and heterosexual experiences among adolescents

There were statistically significant differences in opinions on condom use and sexual experiences by gender (Tables 2 and 3). Specifically, a higher proportion of adolescent boys (89.9%) than adolescent girls (67%) agreed that condoms are an effective way to protect against HIV/AIDS [X^2^ = 13.73; p = 0.001]. Most adolescent girls (78%) believed that it would be too embarrassing to buy condoms compared with 62% of adolescent boys [X^2^ = 6.28; p = 0.043]. Regarding sexual experiences, 30.3% of adolescent girls admitted to having ever used contraceptives compared with 10.1% of boys [X^2^ = 10.90; p = 0.001]. Approximately 10% of adolescent girls aged 15–19 years were forced into having sex compared to their male counterparts, none of whom admitted being forced [X^2^ = 8.47; p = 0.004]. Furthermore, a higher proportion of adolescent girls aged 15–19 years (28.4%) indicated that they had been sexually harassed as compared to 12.7% of adolescent boys [X^2^ = 6.69; p = 0.010]. A higher proportion of adolescent boys aged (39.2%) felt not being pressured to have sex relative to only 9.2% of girl [X^2^ = 24.28; p = 0.000].

**Table 2:** Association between gender, age and opinions on condom use among adolescents (10–19 years). Source: IDRC Adolescent Health Intervention Project, 2019

	**Opinions on Condom use**						
	Agree		Disagree		Don’t know		P-value
	N	%	N	%	N	%	
Condoms are an effective method of preventing pregnancy							
Gender							
Boys	73	92.4	3	3.8	3	3.8	0.496
Girls	95	87.2	6	5.5	8	7.3	
χ^2^ = 1.40							
Age							
10–14	59	78.7	5	6.7	11	14.7	0.000
15–19	109	96.5	4	3.5	0	0.0	
χ^2^ = 19.09***							
A condom can be used more than once							
Boys	7	8.9	58	73.4	14	17.7	0.813
Girls	7	6.4	83	76.1	19	17.4	
χ^2^ = 0.41							
Age							
10–14	9	12.0	43	57.3	23	30.7	0.000
15–19	5	4.4	98	86.7	10	8.8	
χ^2^ = 20.89***							
A girl can suggest to her boyfriend that he uses a condom							
Boys	65	82.3	8	10.1	6	7.6	0.068
Girls	83	76.1	6	5.5	20	18.3	
χ^2^ = 5.36							
Age							
10–14	44	58.7	8	10.7	23	30.7	0.000
15–19	104	92.0	6	5.3	3	2.7	
χ^2^ = 33.69***							
A boy can suggest to his girlfriend that he uses a condom							
Boys	70	88.6	3	3.8	6	7.6	0.337
Girls	88	80.7	6	5.5	15	13.8	
χ^2^ = 2.18							
Age							
10–14	48	64.0	7	9.3	20	26.7	0.000
15–19	110	97.3	2	1.8	1	0.9	
χ^2^ = 38.18***							
Condoms are an effective way of protecting against HIV/AIDS							
Boys	71	89.9	2	2.5	6	7.6	0.001
Girls	73	67.0	14	12.8	22	20.2	
χ^2^ = 13.73**							
Age							
10–14	48	64.0	7	9.3	20	26.7	0.000
15–19	96	85.0	9	8.0	8	7.1	
χ^2^ = 14.30***							
It would be too embarrassing for someone like me to buy or obtain condoms							
Boys	49	62.0	25	31.6	5	6.3	0.043
Girls	85	78.0	18	16.5	6	5.5	
χ^2^ = 6.28*							
Age							
10–14	62	82.7	6	8.0	7	9.3	0.000
15–19	72	63.7	37	32.7	4	3.5	
χ^2^ = 16.92***							
If a girl suggested using condoms to her partner, it would mean that she didn’t trust him							
Boys	33	41.8	31	39.2	15	19.0	0.246
Girls	37	33.9	40	36.7	32	29.4	
χ^2^ = 2.80							
Age							
10–14	27	36.0	15	20.0	33	44.0	0.000
15–19	43	38.1	56	49.6	14	12.4	
χ^2^ = 28.50***							
Condoms reduce sexual pleasure							
Boys	20	25.3	18	22.8	41	51.9	0.626
Girls	25	22.9	20	18.3	64	58.7	
χ^2^ = 0.94							
Age							
10–14	7	9.3	10	13.3	58	77.3	0.000
15–19	38	33.6	28	24.8	47	41.6	
χ^2^ = 24.35***							
Condoms can slip off the man and disappear inside the woman’s body							
Boys	23	29.1	22	27.8	34	43.0	0.521
Girls	28	25.7	25	22.9	56	51.4	
χ^2^ = 1.31							
Age							
10–14	14	18.7	15	20.0	46	61.3	0.010
15–19	37	32.7	32	28.3	44	38.9	
χ^2^ = 9.4**							
If unmarried couples want to have sexual intercourse before marriage, they should use condoms							
Boys	64	81.0	8	10.1	7	8.9	0.512
Girls	81	74..3	13	11.9	15	13.8	
χ^2^ = 1.34							
Age							
10–14	46	61.3	12	16.0	17	22.7	0.000
15–19	99	87.6	9	8.0	5	4.4	
χ^2^ = 19.46***							

^*^P < 0.05, ^**^P < 0.01, ***P < *0.001*

**Table 3:** Association between gender, age and opinions on heterosexual experiences among adolescents (10–19 years). Source: IDRC Adolescent Health Intervention Project, 2019

	Heterosexual Experiences				
Physical Contact-Kissing and hugging					P-value
	No		Yes		
	N	%	N	%	
Gender					
Boys	61	77.2	18	22.8	P = 0.056
Girls	70	64.2	39	35.8	
χ^2^ = 3.66					
Age					
10–14	67	89.3	8	10.7	P = 0.000
15–19	64	56.6	49	43.4	
χ^2^ = 22.81***					
Transactional sex-pay money for sex					
Boys	77	97.5	2	2.5	
Girls	102	93.6	7	6.4	P = 0.217
χ^2^ = 1.52					
Age					
10–14	75	100.0	0	0.0	P = 0.010
15–19	104	92.0	9	8.0	
χ^2^ = 6.27*					
Transactional sex-receive money or gifts for sex					
Boys	76	96.2	3	3.8	
Girls	99	90.8	10	9.2	P = 0.151
χ^2^ = 2.06					
Age					
10–14	74	98.7	1	1.3	P = 0.014
15–19	101	89.4	12	10.6	
χ^2^ = 6.04*					
Adolescent pressured to have sex					
Boys	31	39.2	48	60.8	
Girls	10	9.2	99	90.8	P = 0.000
χ^2^ = 24.28***					
Age					
10–14	12	16.0	63	84.0	P = 0.116
15–19	29	25.7	84	74.3	
χ^2^ = 2.47					
Sexually Coerced					
Boys	79	100.0	0	0.0	
Girls	98	89.9	11	10.1	P = 0.004
χ^2^ = 8.47**					
Age					
10–14	71	94.7	4	5.3	
15–19	106	93.8	7	6.2	P = 0.805
χ^2^ = 0.061					
Sexually harassed					
Boys	69	87.3	10	12.7	
Girls	78	71.6	31	28.4	P = 0.010
χ^2^ = 6.69*					
Age					
10–14	64	85.3	11	14.7	
15–19	83	73.5	30	26.5	P = 0.053
χ^2^ = 3.73					
Ever use of all forms of contraceptives					
Boys	71	89.9	8	10.1	
Girls	76	69.7	33	30.3	P = 0.001
χ^2^ = 10.90**					
Age					
10–14	68	90.7	7	9.3	P = 0.000
15–19	79	69.9	34	30.1	
χ^2^ = 11.39***					

^*^P < 0.05, ^**^P < 0.01, ^***^P < 0.001

### Factors influencing adolescents’ opinion on condoms use and heterosexual experiences

Table 4 shows the results of the binary logistic regression analysis. Being a male or female was a significant influencer of adolescent’s opinion on condom use and heterosexual experiences. Compared to males, adolescent females are 84% less likely to agree with the opinion that condoms are effective means of preventing HIV/AIDS [aOR = 0.16; CI 0.06–0.43]. The result further indicated that adolescents aged 10–14 years have lower odds (57% less) of agreeing to the opinion that condoms are effective ways of preventing HIV/AIDS compared to adolescents aged 15–19 years [aOR = 0.43; CI 0.19–0.99].

**Table 4:** Binary Logistics Regression model of factors influencing adolescents opinion on condom use and heterosexual experiences. Source: IDRC Adolescent Health Intervention Project, 2019

Variables	Opinion on Condom use (effective way of preventing HIV)	Opinion on condom use (embarrassing to purchase condom)	Heterosexual experiences (pressured into sex)
Factors	Model I aOR [95%CI]	Model II aOR [95% CI]	Model III aOR [95% CI]
Gender			
Male	Ref	Ref	Ref
Female	0.16[0.06–0.43]***	2.85[1.33–6.14]**	0.15[0.06–0.39]***
Age			
10–14	0.43[0.19–0.99]*	2.91[1.28–6.63]*	0.78 [0.30–2.06]
15–19	Ref	Ref	Ref
Educational Level			
No Education	Ref	Ref	Ref
Primary	0.56 [0.19–1.66]	0.84 [0.27–2.61]	0.31 [0.09–1.07]
Secondary	1.89 [0.56–6.45]	1.01 [0.31–3.32]	0.52 [0.15–1.87]
Living Arrangement			
Living with only father	0.82 [0.28–2.42]	0.51[0.19–1.36]	1.16 [0.38–3.53]
Living with only mother	2.11 [0.85–5.19]	0.54 [0.24–1.22]	1.40 [0.55–3.59]
Living with both parent	Ref	Ref	Ref
Adolescent Club Member			
No	1.01 [0.42–2.43]	0.56 [0.27–1.19]	0.72 [0.30–1.73]
Yes	*Ref*	Ref	Ref
Social Event Attendance			
No	0.59[0.27–1.31]	0.86[0.42–1.78]	0.44 [0.18–1.07]
Yes	*Ref*	Ref	Ref
Employment Status			
Not employed	*Ref*	Ref	Ref
Employed	0.62[0.10–3.84]	5.90 [1.15–30.34]*	2.77 [0.72–10.68]
Discussed sexual issues with Father			
No	2.09 [0.67–6.52]	1.71[0.61–4.80]	0.80 [0.30–2.15]
Yes	*Ref*	Ref	Ref
Discussed sexual issues with Mother			
No	0.40 [0.16–1.01]	1.50 [0.65–3.47]	0.82 [0.24–2.85]
Yes	*Ref*	Ref	Ref

^*^P < 0.05, ^**^P < 0.01, ***P < 0.001, Ref = Reference category

Furthermore, the relevance of gender in influencing the opinions of adolescents concerning condoms use has been found in this study. Compared to males, adolescent females were 2.85 times more likely to agree to the opinion that it would be too embarrassing for them to go and purchase condoms [aOR = 2.85; CI 1.33–6.14). Adolescents aged 10–14 years are 2.91 times more likely to agree to the opinion that purchasing condoms would be an embarrassing experience for them compared to adolescents aged 15–19 years.

The employment status of adolescents positively influenced their opinions on condom use. Adolescents who are currently employed (work for pay) have higher probability [aOR = 5.90; CI 1.15–30.34] of agreeing with the opinion that it would be embarrassing for them to go and purchase condoms compared to those who are unemployed. Again, female adolescents are 85% less likely to agree with the opinion that they have been pressured into having sexual intercourse compared to males [aOR = 0.15; CI 0.06–0.39].

## Discussion

This paper examined gender dynamics and socio-cultural factors that influence opinions on condom use and sexual experiences of adolescents in a high-adolescent pregnancy setting in Adaklu district in the Volta Region of Ghana. The educational level, living arrangement, social club membership, attendance of social events and adolescent communication on sexual issues with their parents were used as proxies to measure social and cultural factors. The results show that adolescent girls have a conservative opinion about condom use, whereas adolescent boys have liberal opinions on condom use. Adolescent girls were less likely to agree with the opinion that condom is an effective way of preventing HIV/AIDS compared to boys. This finding concurs with earlier studies [12, 13, 15], where societal pressure to conform to traditional gender roles may make it more challenging for females to negotiate condom use with their male partners. These studies found that females are more likely to have negative sexual experiences, including sexual coercion and unwanted sexual encounters, than males. These negative experiences may impact their attitudes toward condom use and make it more challenging for them to negotiate condom use with their male partners. This may reflect differences in the level of control that males have about the use of condoms regarding negotiations about sexual intercourse. Socio-cultural and gender norms may stigmatize condom use, leading to negative attitudes toward condoms and a decreased likelihood of use among adolescent boys and girls [26–29]. Specifically, this study found that a higher proportion of adolescent boys agreed with the opinion that condoms are an effective way to protect against HIV/AIDS compared to adolescent girls. A similar finding was reported by an earlier study in Burkina Faso and Ghana by Guiella and Madise [41] and Krugu and colleagues [42], respectively. These studies found that adolescent boys were more likely to mention HIV prevention compared to adolescent girls. They further found that most adolescent girls cited pregnancy prevention as the reason for condom use. This indicates that adolescent girls are more concerned about pregnancy, whereas adolescent boys are more concerned about preventing STDs and HIV/AIDS.

Gender norms and expectations influence adolescent sexual behaviours and attitudes towards condom use [27, 28]. Our study findings show that most adolescent girls believe that it would be too embarrassing to buy condoms compared with adolescent boys. Also in the regression analysis, the study found that adolescent girls are more likely to agree with the opinion that it would be embarrassing for them to go and purchase condoms at the pharmacy compared to adolescent boys. Earlier studies have also reported similar findings [14, 24], where adolescent boys exhibit confidence when they go to the pharmacy to buy condoms. This clearly implies that society perceives females as promiscuous if they purchase a condom, and this negative labelling affects the social and cognitive wellbeing of females, especially in patriarchal societies [43–45]. This may affect the negotiation power of females concerning safe and protected sexual intercourse and increase their vulnerability to unplanned pregnancies and sexually transmitted infections.

Furthermore, the age of adolescents has been found to influence their opinion on condom use [46–48]. These studies found that adolescents aged below 16 years had conservative attitude and behaviour towards condoms and a low condom use prevalence. Our study found that adolescents aged 10–14 years are less likely to agree with the opinion that condoms are an effective way of preventing HIV compared to those aged 15–19 years. Further, adolescents aged 10–14 years are more likely to agree to the opinion that it will be embarrassing for them to go and purchase condoms compared to adolescents aged 15–19 years. The findings of this study concur with those of earlier studies [46–58], and the possible explanation for this occurrence may be as a result of limited sexual education where adolescents aged 10–14 may have received less comprehensive sexual education compared to those aged 15–19. This lack of information about the correct use and effectiveness of condoms can contribute to misconceptions. Also, adolescents aged 15–19 are more likely to be sexually active compared to those aged 10–14. This increased sexual activity may lead to a greater understanding of the importance and effectiveness of condoms in preventing sexually transmitted infections, including HIV and pregnancy.

Our study found that adolescents who are currently employed (working for pay) have a higher probability to agree with the opinion that it would be embarrassing for them to purchase condoms compared to those who are not currently employed. Probably, employed adolescents may fear social stigma from colleagues or superiors if they are seen purchasing condoms [52, 54]. In some work environments or cultures, discussing or acknowledging sexual health openly may be considered inappropriate, leading to embarrassment. Also, adolescents in the workforce may be particularly sensitive to how they are perceived professionally. Purchasing condoms may be seen as a marker of sexual activity, and some employed adolescents may be concerned about the impact this could have on their professional image.

Adolescent boys and girls have different heterosexual experiences as documented by previous studies in varied settings [26, 42, 46–49]. The study findings show adolescent girls are less likely to agree with the opinion that they have been pressured into sexual intercourse compared to boys. The possible explanation for this is that, social expectations and gender norms often influence behaviour. Girls may be socialised to be more compliant, nurturing and accommodating, which could affect their willingness to report feeling pressured. Boys, on the other hand, may feel more pressure to conform to stereotypical masculine behaviour, leading them to be more vocal about perceived pressure. Again, power dynamics in relationships can play a role. Girls may feel less empowered to resist pressure, especially in situations where there is an imbalance of power and influence. Boys might be more accustomed to asserting themselves or may perceive that they have more agency to resist pressure.

The study findings show a higher proportion of girls reported ever using contraception compared to boys. Studies have argued that females are concerned about the prevention of pregnancy and may use various forms of contraceptives to achieve that [41–44]. Adolescent girls with multiple male partners often use more contraceptives to avoid pregnancy and sexually transmitted infections; this explains the higher contraceptive use among girls compared to boys [43, 44]. Other reasons include the stigmatization associated with teenage pregnancy among girls, especially in traditional societies, which compelled them to use contraception to avoid the embarrassment and community shaming of teenage pregnancy.

Studies have shown a high proportion of adolescent girls experiencing sexual coercion compared with boys [53–59]. Our study findings revealed that relatively higher proportion of girls reported being coerced into engaging in sexual activities whereas none of the boys reported being sexually coerced. These results suggest that females are usually more vulnerable to sexual exploitation compared with males and are often forced to engage in sexual activities against their wishes in a highly male – dominated society. Similarly, the study found that a higher proportion of adolescent girls were sexually harassed compared with boys. This also reemphasizes the sexual vulnerabilities and victimisation of adolescent girls as well as the social perception of girls as “*sexual objects*” as reported by other studies [60–63]. These studies further revealed that adolescent girls tend to have negative cognitive, psychological and physical development because of these negative sexual experiences compared with boys.

## Strengths and limitations of the study

This study contributes to knowledge gap by highlight the gender dynamics and socio-cultural factors that influence the opinions on condom use and sexual experiences of adolescents in a high-adolescent pregnancy setting. The findings of this study also provide useful insight that will help Ghana’s Adolescent Health Policy and strategy in filling implementation gaps especially on gender sensitive issues and SRH needs for adolescent boys and girls.

However, the study had some limitations: first the study relayed on quantitative data, so we could not explore other social and cultural beliefs and practices that might influence adolescent opinion on condom use and their heterosexual experiences. Again, there is a likelihood of adolescents giving socially desirable responses due to the sensitive nature of the subject matter under study.

## Conclusions

This study contributes to knowledge by highlighting gender differences and socio-cultural factors influencing opinions on condom use and sexual experiences among adolescents in a high teenage pregnancy setting in rural Ghana. Specifically, the results reveal that adolescent girls are more likely to perceive buying a condom as embarrassing and less likely to agree with the opinion that condoms are an effective way for HIV prevention relative to boys. This implies a lack of sexual bargaining power on the part of girls in terms of condom usage as they are unable to purchase condoms, resulting from a combination of limited access to resources, conservative social and cultural norms with regards to premarital sexual relations [38]. These root drivers perpetuate the limited agency of adolescent girls in their sexual health and relations, which can give rise to a sense of negativity, gender stereotyping and the injustice of the sexist value system that affects girls from adolescents throughout their lifetime. This also places females in a sexually vulnerable position, which could result in negative sexual outcomes such as rapes and other forms of sexual harassment that could result into teenage pregnancies and the acquisition of sexually transmitted diseases. Also, the present study found that a higher proportion of adolescent girls were pressured to have sexual intercourse and are sexually harassed compared to adolescent boys. The gender of an adolescent is critical to their sexual and reproductive health and wellbeing as the study findings show distinct sexual experiences of adolescent boys and girls. This further implies, no one size intervention will suit all males and females in the study. Thus, at the intervention phase, boys or girls were engaged separately to identify interventions that will empower boys and girls to make informed SRH decisions, have autonomy and be confident about their SRH rights. The outcome of these interventions and engagements along with the endline results of will be published later.

## Supplementary Information

Additional file 1.

## Data Availability

The data and all materials analysed/used for this study will be made available and accessed upon reasonable request through the corresponding author to the Principal Investigator.

## Abbreviations

HIV/ AIDS: Human immunodeficiency virus/ acquired immunodeficiency syndrome
REC: Research Ethics Committee
STDs: Sexually transmitted diseases
UHAS: University of Health and Allied Sciences
