# Supplementary material for: Gender differences and factors affecting opinions on condom use and sexual experiences among adolescents in a high teenage pregnancy setting in the Volta Region, Ghana
Source: Reprod Health. 2025 May 31;22(Suppl 1):76. doi: 10.1186/s12978-025-01990-7 (PMC12125764; doi:10.1186/s12978-025-01990-7)
Supplement: Supplementary file 1 — Additional file 1. [file 12978_2025_1990_MOESM1_ESM.docx]

**BMC Supplements**

Submission ID: **SUPP-D-24-00099R1**

Manuscript title**:** **Gender differences and factors affecting opinions on condom use and sexual experiences among adolescents in a high teenage pregnancy setting in the Volta Region, Ghana**

**Point by point response to reviewer’s file**

| **Response to Reviewer comments** | | |
| --- | --- | --- |
| **S/N** | **Comments** | **Response** |
| 1 | As written this implies there are 37,904 households, of 3.7 people each, which would make for a population of 140,244.80 people. Yet earlier you state that the population of the district is 38,649. I would suggest you rephrase as this sentence to remove the total household figure, and only mention the average household size. See suggested edit. | This has been rephrase as suggested. Kindly refer first paragraph under “***study area and period section***” on page **7** |
